# Vaccine Effectiveness of Polysaccharide Vaccines Against Clinical Meningitis - Niamey, Niger, June 2015

**DOI:** 10.1371/currents.outbreaks.5d6e9c1d071a2088109c242771b68886

**Published:** 2016-04-18

**Authors:** Marc Rondy, Djibo Issifou, Alkassoum S. Ibrahim, Zaneidou Maman, Goumbi Kadade, Hamidou Omou, Sidikou Fati, Esther Kissling, Sarah Meyer, Olivier Ronveaux

**Affiliations:** EpiConcept, Paris, France; Niamey II Health District, Ministry of Health, Niamey, Niger; Division of Surveillance and Epidemiological Response (DSRE), Ministry of Health, Niamey, Niger; Division of Surveillance and Epidemiological Response (DSRE), Ministry of Health, Niamey, Niger; Division of Surveillance and Epidemiological Response (DSRE), Ministry of Health, Niamey, Niger; World Health Organization, Geneva, Switzerland; Centre de Recherche Médicale et Sanitaire (CERMES), Ministry of Health, Niamey, Niger; EpiConcept, Paris, France; Meningitis and Vaccine Preventable Diseases Branch, Centers for Disease Control and Prevention, Atlanta, Georgia, USA; World Health Organization

## Abstract

Introduction: In 2015, a large outbreak of serogroup C meningococcal meningitis hit Niamey, Niger, in response to which a vaccination campaign was conducted late April. Using a case-control study we measured the vaccine effectiveness (VE) of tri - (ACW) and quadrivalent (ACYW) polysaccharide meningococcal vaccines against clinical meningitis among 2-15 year olds in Niamey II district between April 28th and June 30th 2015.

Methods: We selected all clinical cases registered in health centers and conducted a household- vaccination coverage cluster survey (control group).  We ascertained vaccination from children/parent reports. Using odds of vaccination among controls and cases, we computed VE as 1-(Odds Ratio). To compute VE by day since vaccination, we simulated a density case control design randomly attributing recruitment dates to controls based on case dates of onset (3 controls per case). We calculated the number of days between vaccination and the date of onset/recruitment and computed VE by number of days since vaccination using a cubic-spline model. We repeated this simulated analysis 500 times and calculated the mean VE and the mean lower and upper bound of the 95% confidence interval (CI).

Results: Among 523 cases and 1800 controls, 57% and 92% were vaccinated respectively. Overall, VE at more than 10 days following vaccination was 84% (95%CI: 75-89) and 97% (94-99) for the tri- and quadrivalent vaccines respectively. VE at days 5 and 10 after trivalent vaccination was 84% (95% CI: 74-91) and 89% (95% CI: 83-93) respectively. It was 88% (95% CI: 75-94) and 95.8% (95% CI: 92 -98) respectively for the quadrivalent vaccine.

Conclusion: Results suggest a high VE of the polysaccharide vaccines against clinical meningitis, an outcome of low specificity, and a rapid protection after vaccination. We identified no potential biases leading to VE overestimation. Measuring VE and rapidity of protection against laboratory confirmed meningococcal meningitis is needed.

## Background

In the meningitis belt of sub-Saharan Africa, meningococcal meningitis epidemics have historically been due to serogroup A Neisseria meningitidis and more recently due to serogroups W and X^1^. In the four years following mass vaccination campaigns with a novel serogroup A meningococcal conjugate vaccine (MACV, MenAfriVac™) in Niger in 2010 and 2011^2^, no meningitis epidemics were observed. However, in 2015, the first large-scale epidemic in Africa due to serogroup C Neisseria meningitids (NmC), a rare cause of meningitis in the region, was reported in Niger with over 8,500 suspected cases^3^. The urban district of Niamey II, with 2,615 suspected meningitis cases, was particularly hard-hit.

In response to this epidemic, Niamey II district organized mass school-based vaccination campaigns using trivalent (ACW)^4^ and quadrivalent (ACYW)^5^ polysaccharide vaccines from April 24th to 30th 2015. Quadrivalent polysaccharide vaccines were also distributed at health centers and at the district chief’s dwelling during the same time period. Due to a global shortage of polysaccharide meningococcal vaccines, vaccination campaigns were limited to children aged 2-15 years.

Very little is known in terms of vaccine effectiveness (VE) of polysaccharide meningococcal vaccines against NmC in Africa. Compared to developed regions, VE in the African context may differ according to differences in host responses, natural or acquired immunity^6^, and vaccine delivery issues such as inadequate storage and transport^7^. There is also a lack of data regarding the time needed to acquire protection after vaccination. Such information would be useful for planning meningococcal vaccination campaigns during epidemics.

In order to guide the response to future meningococcal epidemics, we measured the VE of polysaccharide meningococcal vaccines against clinical meningitis among children aged 2-15 years in the district of Niamey II, Niger, during the period of April 28th-June 30th, 2015. We stratified the VE by age group (children aged 2-5, 6-10 and 11-15 years) to detect potential age group-specific differences in VE. We computed the VE by day since vaccination.

## Methods

**Study design**

We conducted a case-control study^8^, comparing the odds of vaccination among suspected meningitis cases to controls enrolled in a vaccine coverage survey performed at the end of the epidemic. The study was conducted in collaboration with the Ministry of Health after obtaining permission to conduct the survey. The study protocol was approved by the National Ethical Review Board of Niger. Informed oral consent was obtained from all study participants' legal tutors.

**Study population**

We defined the study population as any child aged 2-15 years residing in the district II of Niamey, Niger, at any time between April 28th and June 30th 2015. Children away from the district II of Niamey during the vaccination campaign (April 24th-30th) were excluded from the study.

**Case definition**

We used the clinical case definition (suspected case according to WHO guidelines^9^) implemented at the health center triage: sudden onset of fever (> 38 ° C axillary) and at least one of the following: neck stiffness, impaired consciousness, or other meningeal signs.

**Vaccination status ascertainment**

We collected vaccination status as well as place and date of vaccination through face-to-face interviews with the child’s parents. As no vaccination cards were distributed during the campaigns and no vaccine registers were maintained, a child was considered to be vaccinated if the child or their parent reported them to have received a vaccine against meningitis between April 1st and June 30th, 2015. The regional health authorities provided the list of vaccination sites with the type of vaccine (trivalent or quadrivalent) administered per site. Comparing this list with the childs’s place of vaccination, we identified which vaccine type was administered.

**Recruitment procedures**

From June 30th to July 10th, 2015, we reviewed line lists at the districts health centers to recruit suspected meningitis cases admitted between April 28th and June 30th. Through community health workers, we contacted and interviewed the consenting cases’ parent or guardian.

We conducted a vaccine coverage survey using EPI cluster sampling methodology^10^ to measure vaccine uptake after the campaign. As this survey was a representative sample of the population at risk, the source of the meningitis cases, we used it as the control group for our case control study. We chose this method to obtain a sample geographically representative of the district while keeping the survey logistically feasible. We conducted the survey from June 26th to June 30th 2015 among 2-15 years old children residing in the district of Niamey II. We calculated a sample size of at least 70 cases and 560 controls to estimate a VE of 90% with 5% precision, assuming 80% coverage in the source population and a design effect of 2. We selected sixty clusters of ten individuals in each age group (2-5, 6-10 and 11-15 years), randomly selecting one child per age group from the household. We used random GPS coordinates to identify the first household in the cluster, with the closest neighboring households then invited to participate.

**Data collection**

For each child, we collected information on age, sex, vaccination date, vaccination site, district of residence, and place of school attendance at the time of the school-based vaccination campaigns. For cases, we also collected the dates of symptom onset and health center admission. For cases with missing symptom onset dates, we imputed the value based on their date of admission and the median delay between onset and admission among children with complete data.

**Data analysis**

We weighted vaccine coverage measures using the number of eligible children by age group in the household (weight of 1 assigned to the cases) and took into account the cluster design effect. The presence of sex and school attendance as confounding factors was investigated and VE estimates adjusted accordingly.

**Overall Vaccine Effectiveness**

Assuming development of protective antibodies against meningococcal disease by 10 days after vaccination^11^, we excluded cases with onset less or equal to 10 days after vaccine receipt. To ensure the same probability of exposure to the vaccination campaign for cases and controls, we excluded cases with date of onset before May 10th (end of vaccination campaign on April 30th + 10 days). We calculated an odds ratio (OR) comparing the odds of vaccination among cases and controls. We calculated the VE as (1-OR) * 100. We calculated the overall VE for polysaccharide meningococcal vaccines as well as trivalent and quadrivalent-specific VE. We stratified the analyses by age group.

**Vaccine Effectiveness by time since vaccination**

We simulated a density case control study^8^ using all cases reported from April 28th to June 30th. We randomly allocated recruitment dates to controls based on cases’ dates of onset (three controls per case). We calculated the number of days between vaccination and the simulated date of onset/recruitment. We computed VE by number of days since vaccination using a cubic-spline model^12^. For this analysis, we also included cases and controls vaccinated less than or equal to 10 days prior to date of onset/recruitment. We repeated these simulations 500 times and presented the mean VE and the mean lower and upper bounds of the 95% confidence interval. All simulated density case-control analyses were adjusted by date of onset/recruitment.

**Laboratory data**

Cerebrospinal fluid (CSF) was collected on suspected meningitis cases through lumbar puncture. The CSF were analyzed by PCR and culture for Neisseria meningitidis (including serogroup), Haemophilus influenzae, and Streptococcus pneumoniae at the national reference laboratory for meningitis. We compared line lists from the laboratory and health centers to match laboratory results with clinical cases.

## Results

We included 1,800 children in the control group recruited through the cluster sampling survey. The overall vaccination coverage was 91.6%. The vaccine coverage was 89.5%, 94.0% and 90.9% among children aged 2-5 years, 6-10 years and 11-15 years respectively. Among vaccinated children, 51.9% received the trivalent vaccine and 32.8% the quadrivalent. We were unable to determine the type of vaccine received in 15.2%.

**Figure 1:**
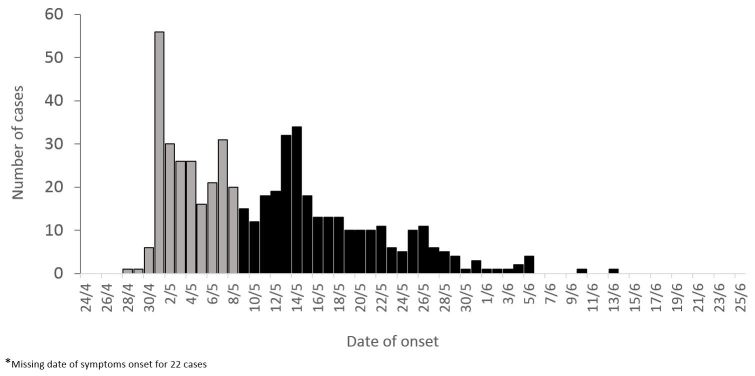
**Figure 1:** Clinical meningitis cases (N=523) among children aged 2-15 years by date of symptoms onset, Niamey II, Niger, April-June 2015

From the health center registries, we identified 572 clinical cases of meningitis and contacted 523. They occurred between April 28th and June 13th, peaking during the first two weeks of May before slowly decreasing until early June (Figure 1).

Of these 523 clinical cases, we found 58 matching laboratory results. Among them, 43 were negative, one undetermined, one positive for S. pneumoniae and 14 positive for Neisseria meningitidis, including 12 NmC. Given the low number of laboratory confirmed cases, we were unable to conduct meningococcal or serogroup-specific analyses.

**Figure 2:**
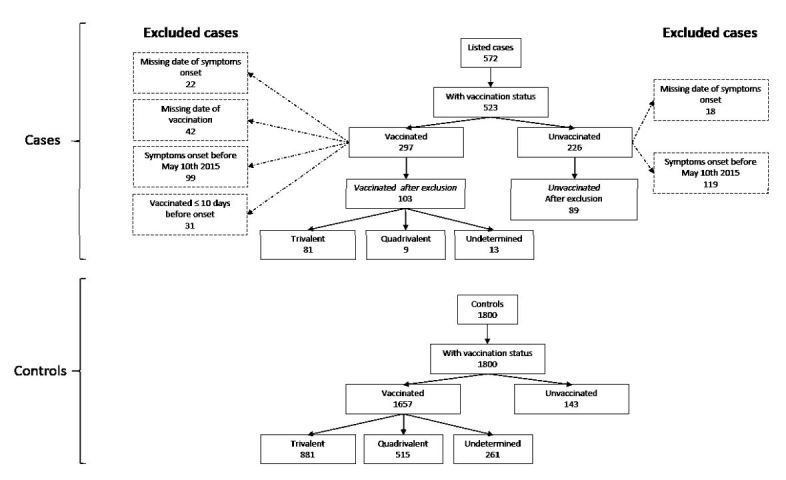
**Figure 2** **:** Exclusion flowchart of cases of clinical meningitis and controls, Niamey II, Niger, April-June 2015

After excluding cases with missing dates of symptom onset (N=40) or vaccination (N=42), onset before May 10th (N=218), and those vaccinated within 10 days before symptoms onset (N=31), we included 192 cases in the primary analysis (Figure 2).

Of them, 103 (53.6%) were vaccinated. Among the vaccinated cases, 78.6%, 8.7% and 12.6% received the trivalent, quadrivalent and an undetermined vaccine respectively. The vaccine coverage was 41.2%, 73.0% and 46.2% among cases aged 2-5 years, 6-10 years and 11-15 years respectively.

The overall VE against clinical meningitis at more than 10 days after vaccination was 89.3% (95% CI: 84.3-92.8). It was 83.9% (95% CI: 75.4-89.4) for the trivalent vaccine and 97.2% (95% CI: 94.0-98.7) for the quadrivalent vaccine (Table 1).

**Figure:**
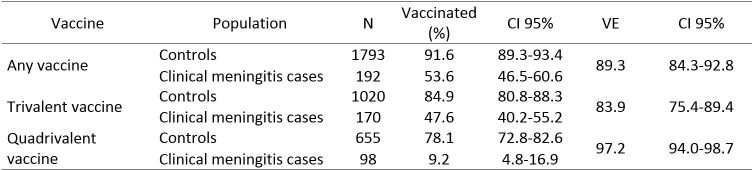
**Table 1:** Polysaccharide vaccine effectiveness against clinical meningitis by vaccine type among children aged 2-15 years, Niamey II, Niger, April-June 2015

The VE did not vary by age group (Table 2).

**Figure:**
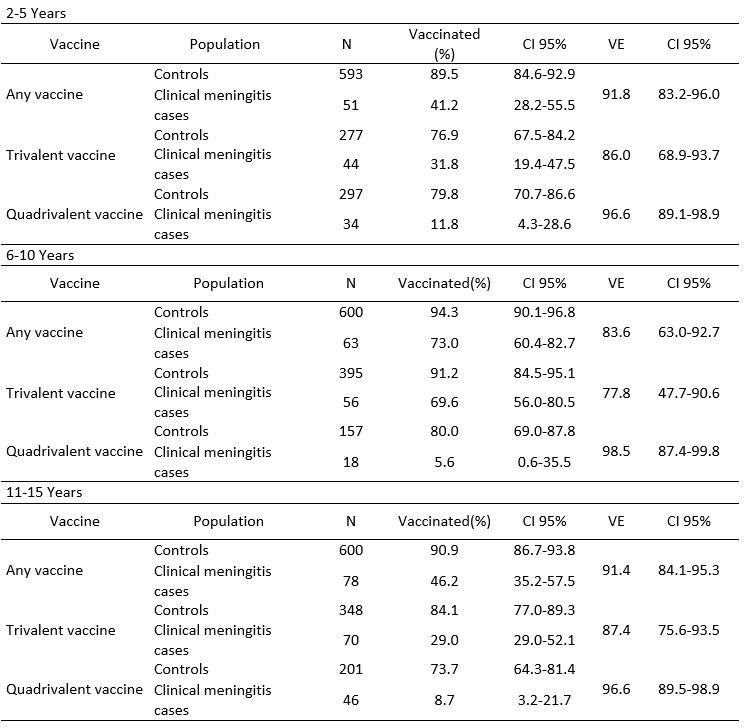
**Table 2:** Polysaccharide vaccine effectiveness against clinical meningitis by vaccine type and age group, Niamey II, Niger, April-June 2015

Adjustment for age, sex or school attendance did not change the VE.

**Figure 3:**
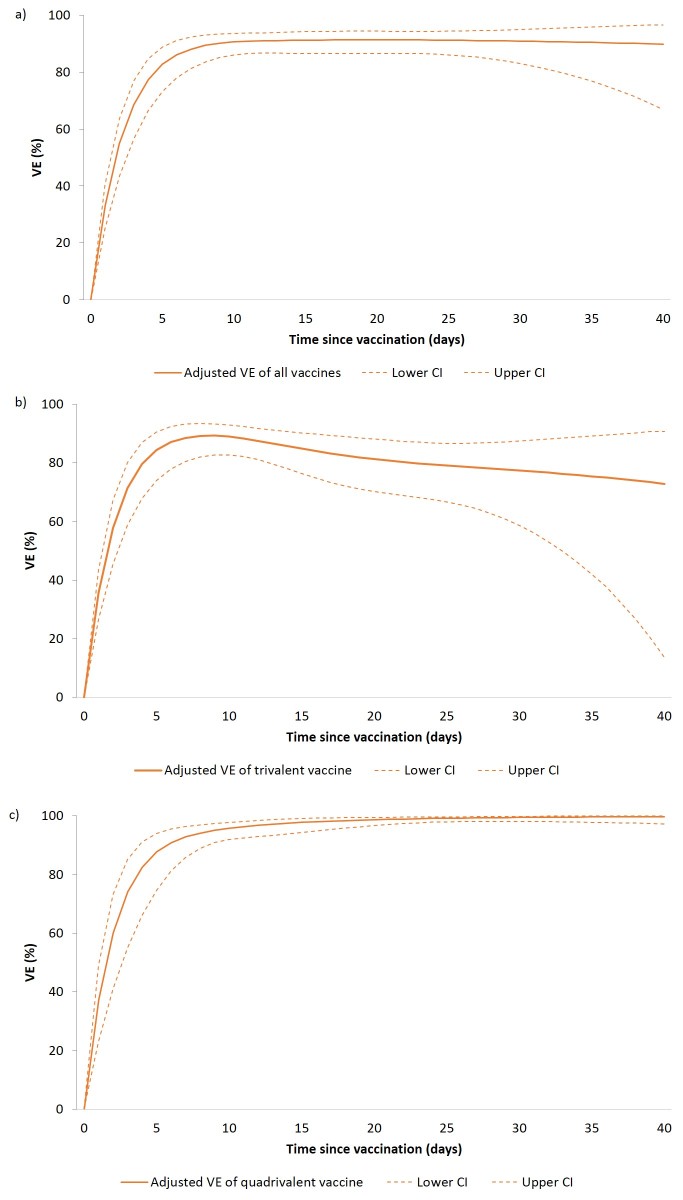
**Figure 3:** Adjusted vaccine effectiveness of any polysaccharide vaccine (a), trivalent polysaccharide vaccine (b) and quadrivalent polysaccharide vaccine (c) against clinical meningitis by vaccine type and by time since vaccination among children aged 2-15 years, Niamey II, Niger, April-June 2015

Using a simulated density case-control approach, we computed VE by time since vaccination for all cases, including those with onset within 10 days of vaccination.

For the trivalent vaccine, VE at five and ten days after vaccination were 84.4% (95% CI: 74.0-90.5) and 89.0% (95% CI: 82.7-92.9) respectively. For the quadrivalent vaccine VE at five and ten days after vaccination were 87.7% (95% CI: 74.6-94.0) and 95.8% (95% CI: 92.0-97.8) respectively (Figure 3).During this NmC meningitis epidemic in Niger, the effectiveness of polysaccharide vaccines against clinical meningitis in 2-15 year olds was high at 89.3% overall effectiveness, with 97.2% effectiveness of the quadrivalent vaccine and 83.9% effectiveness of the trivalent vaccine. Although some prior evaluations suggest age group-dependent vaccine effectiveness^13^, with lower VE in young children, we observed high VE in all age groups of children aged 2-15 years.

## Discussion

Our simulated results suggest a fast increase of the VE after vaccination, reaching more than 80% after five days and 90% after ten days. Such high VE combined with the very high vaccine coverage measured in Niamey may have contributed to the rapidly decreasing incidence following the vaccination campaign. However, based on prior evaluations, polysaccharide vaccines provide a short duration of protection^6^^,^ 14 ^,^ 15 and do not impact nasopharyngeal carriage^16^, making them unsuitable for preventive vaccination campaigns.

We observed a higher VE of the quadrivalent compared to the trivalent vaccine. This difference cannot be explained by the valences included in the vaccines as no NmY was isolated during the study period in Niamey. Further investigations, based on individual bacteriologic and vaccination data, are needed to understand these differences.

This observational case control study presents a number of limitations. Only 14 (2.7%) of the clinical cases recruited in our evaluation were laboratory-confirmed. Due to this low number and the uncertainty in terms of representativeness of these cases regarding vaccination status, we decided not to compute VE against laboratory confirmed results. Lack of specificity in the suspected meningitis case definition used may have led to inclusion of cases due to other etiologies, leading to under-estimation of the VE. However, based on results in all age groups during the entire epidemic period, of the 1,456 specimens positive for any bacterial pathogens at the national reference laboratory, 92.6% were positive for N. meningitidis; 1,087 (74.7%) for group C and 196 (13.5%) for serogroup W^3^, suggesting a very high proportion of N. meningitidis covered by the polysaccharide vaccines among laboratory-confirmed cases. Furthermore, our VE estimates are in line with previously published studies conducted in the USA^17^, Spain^18^ and Canada^13^ using laboratory outcome.

We cannot rule out that some medical staff may have been influenced by the vaccination status of the patients in their clinical diagnoses. If so, vaccinated clinical cases may have been excluded as a suspected meningitis case, leading to an overestimation of the VE. However the absence of systematic collection of vaccination status upon patient presentation makes this selection bias unlikely to have distorted the results.

Recruiting controls at the end of the epidemic did not to take into account the changing vaccination coverage during the epidemic. In Niger, the vaccination campaign took place at the peak of the epidemic while cases were still occurring. To account for this changing vaccine coverage in the overall VE estimation, we restricted our analyses to cases with onset more than ten days after the end of the vaccination campaign. With this restriction in the study period, cases and controls had the same opportunity for vaccination. The best approach to account for the changing vaccination coverage over time would have been a prospective, concurrent recruitment of cases and controls through a density case-control design^8^. However, due to logistical constraints of a prospective evaluation, we instead simulated a density case-control evaluation to measure VE by time since vaccination. Although subject to random errors, we believe that such an approach can be useful when limited means do not allow prospective subject recruitment.

In the absence of an affordable, polyvalent conjugate meningococcal vaccine, polysaccharide meningococcal vaccines will continue to play a key role in the response to serogroup C and W meningococcal epidemics in Africa. Strengthening of case-based surveillance for future evaluations of meningococcal and serogroup-specific vaccine effects in Africa will be important to further demonstrate the effectiveness and impact of these vaccines.

## Competing Interests

The authors have declared that no competing interests exist.
